# Modulation of TGF-β/BMP-6 expression and increased levels of circulating smooth muscle progenitor cells in a type I diabetes mouse model

**DOI:** 10.1186/1475-2840-9-55

**Published:** 2010-09-21

**Authors:** Peter E Westerweel, Cindy TJ van Velthoven, Tri Q Nguyen, Krista den Ouden, Dominique PV de Kleijn, Marie Jose Goumans, Roel Goldschmeding, Marianne C Verhaar

**Affiliations:** 1 Department of Nephrology and Hypertension, University Medical Center Utrecht, Utrecht, the Netherlands; 2 Department of Pathology, University Medical Center Utrecht, Utrecht, the Netherlands; 3 Department of Experimental Cardiology, University Medical Center Utrecht, Utrecht, the Netherlands; 4 Department of Molecular Cell Biology, Leiden University Medical Center, Leiden, The Netherlands

## Abstract

**Background:**

Diabetic patients experience exaggerated intimal hyperplasia after endovascular procedures. Recently it has been shown that circulating smooth muscle progenitor cells (SPC) contribute to intimal hyperplasia. We hypothesized that SPC differentiation would be increased in diabetes and focused on modulation of TGF-β/BMP-6 signaling as potential underlying mechanism.

**Methods:**

We isolated SPC from C57Bl/6 mice with streptozotocin-induced diabetes and controls. SPC differentiation was evaluated by immunofluorescent staining for αSMA and collagen Type I. SPC mRNA expression of TGF-β and BMP-6 was quantified using real-time PCR. Intima formation was assessed in cuffed femoral arteries. Homing of bone marrow derived cells to cuffed arterial segments was evaluated in animals transplanted with bone marrow from GFP-transgenic mice.

**Results:**

We observed that SPC differentiation was accelerated and numeric outgrowth increased in diabetic animals (24.6 ± 8.8 vs 8.3 ± 1.9 per HPF after 10 days, p < 0.05). Quantitative real-time PCR showed increased expression of TGF-β and decreased expression of the BMP-6 in diabetic SPC. SPC were MAC-3 positive, indicative of monocytic lineage. Intima formation in cuffed arterial segments was increased in diabetic mice (intima/media ratio 0.68 ± 0.15 vs 0.29 ± 0.06, p < 0.05). In GFP-chimeric mice, bone marrow derived cells were observed in the neointima (4.4 ± 3.3 cells per section) and particularly in the adventitia (43.6 ± 9.3 cells per section). GFP-positive cells were in part MAC-3 positive, but rarely expressed α-SMA.

**Conclusions:**

In conclusion, in a diabetic mouse model, SPC levels are increased and SPC TGF-β/BMP-6 expression is modulated. Altered TGF-β/BMP-6 expression is known to regulate smooth muscle cell differentiation and may facilitate SPC differentiation. This may contribute to exaggerated intimal hyperplasia in diabetes as bone marrow derived cells home to sites of neointima formation.

## Background

Diabetes mellitus greatly increases the risk of cardiovascular disease (CVD) and adversely affects the outcome after endovascular procedures. Diabetic patients experience higher rates of restenosis due to intimal hyperplasia[1,2]. Previously it was thought that accumulation of smooth muscle cells in the neointima of restenotic lesions was exclusively due to migration and local proliferation of medial smooth muscle cells or adventitial fibroblasts. However, it was recently shown in bone marrow chimeric animals that smooth muscle cells of bone marrow origin contribute to postangioplasty restenosis[3]. Consistently, cells with smooth muscle cell characteristics can be isolated from animal[4,5] and human[6-8] blood. These smooth muscle progenitor cells (SPC) may display characteristics of other mesenchymal-lineage phenotypes such as fibroblasts and have also been referred to as circulating 'fibrocytes' or 'myofibroblast progenitor cells'[8,9]. As these cells appear to lack the expression of several specialized smooth muscle proteins such as h-caldesmon and desmin after incorporation, their potential to adopt a phenotype comparable to a mature smooth muscle cell may be limited to a certain extent[10]. We have shown that in Type I diabetic patients, the outgrowth of cells with a smooth muscle/myofibroblast phenotype from cultured mononuclear cells was increased and that BMP-6 expression in these cells was down regulated[9]. Our present study aims to bring further evidence for enhanced SPC differentiation in diabetic conditions in the better-controlled experimental setting of an inducible diabetic mouse model. In addition, we evaluated the effect of diabetes on TGF-β-expression in cultured SPC, as TGF-β is known to counteract BMP-6 signaling and enhance intimal hyperplasia. We hypothesized that inducing Type I diabetes in mice enhances SPC differentiation and numeric outgrowth with decreased BMP-6 expression and increased TGF-β expression in diabetic SPC.

## Methods

### Animals and induction of diabetes

Diabetes was induced in male, eleven week old C57BL/6 mice (Harlan, Horst, the Netherlands) by a single intraperitoneal injection with 200 mg/kg streptozotocin (STZ; Serva, Heidelberg, Germany; n = 11 vs. 9 controls). Insulin-releasing pellets (Linbit, Linshin, Scarborough, Canada) were placed subcutaneously, providing a low insulin dose that is below normal physiological levels and is still associated with marked (more than twice upper limit of normal) hyperglycemicia, but prevents severe catabolism and spontaneous deaths. According to the manufacturer's instructions, 2 insulin-releasing pellets should be given to correct hyperglycemia in a 20-gram diabetic mouse plus 1 pellet for each additional 5 grams body weight. Our animals weighed approximately 25 grams and thus received a third of the recommended insulin dose. Blood glucose levels in samples drawn from conscious animals by tailbleeding were measured using a portable glucose meter (Medisense Precision Xtra; Abbott Laboratories, Bedford, USA). HbA1c was determined in EDTA anti-coagulated blood by HPLC method. All experiments were approved by the local ethics committee on animal experiments.

To enable tracking of bone marrow derived cells *in vivo*, in a separate set of experiments, we transplanted bone marrow (5 × 10^6 ^cells i.v./animal) from GFP-mice (strain C57Bl/6-Tg(UBC-GFP)30Scha/J obtained from the Jackson laboratory, Maine, United States) to lethally irradiated animals (700cGy whole-body γ-irradiation delivered by linear accelerator). Peripheral blood chimerism was evaluated using flowcytometry on peripheral blood leukocytes after lysing erythrocytes with an ammonium chloride lysis buffer. Chimeric animals were required to have at least 90% GFP-positive leukocytes to be included in the experiments.

### Cuff model of intima hyperplasia

Five weeks after onset of diabetes, a non-constrictive polyethylene cuff (0,4 mm inner diameter, 0,8 mm outer diameter, length 2 mm, Portex, Kent, UK) was loosely placed around both femoral arteries. 21 days after cuff placement, cuffed arterial segments were harvested after perfusion with 0.9% saline containing 0.1 mg/ml nitro-glycerine at 120 mm Hg for 5 minutes, fixed in formaldehyde and embedded in paraffin. Serial 5 μm cross-sections were obtained at 200 μm intervals over the length of the cuffed femoral artery segment for histological analysis. 4 equally spaced cross sections of each arterial segment were stained with Elastin Von Gieson staining and the intimal and medial cross-sectional areas were measured using computerized morphometric analysis (Soft Imaging Systems, Münster, Germany). Neointimal smooth muscle cells were identified using biotinylated mouse-anti-human-αSMA antibody (clone 1A4, Sigma) and streptavidin-peroxidase/TRITC-Tyramide Signal Amplification (TSA) system (PerkinElmer, Boston, USA) and counted.

Cryostat sections from neointimal lesions in cuffed arterial segments (n = 18 cuffed segments from 9 animals) and control non-cuffed arterial segments from GFP-chimeric animals were stained with Cy3-conjugated mouse monoclonal anti-αSMA antibody (clone 1A4, Sigma) and DAPI. GFP-positive cells in neointima and adventitia were identified using direct fluorescencence microscopy and the average number of incorporating cells per section was quantified. For detailed evaluation of fluorescence patterns, selected sections were scanned using a confocal fluorescence microscope. To exclude possible misinterpretation by autofluorescence or fluorescence channel bleed-through artefacts, GFP-epifluorescence was confirmed by measuring emission wave length spectrum.

### Smooth muscle progenitor cell culture

SPC were obtained by culturing spleen mononuclear cells on fibronectin-coated dishes in Dulbecco's Modified Eagle Medium (DMEM) supplemented with 20% heat-inactivated fetal calf serum to facilitate smooth muscle cell differentiation. After 4 and 10 days in culture, SPC were identified by immunofluorescent staining for αSMA using biotinylated mouse-anti-human αSMA antibody (Sigma) and streptavidin-peroxidase/TSA system, and staining for collagen type 1 using a goat-anti-human collagen type 1 polyclonal antibody (Southern Biotechnology Associates, Birmingham, USA) and peroxidase labelled rabbit anti-goat immunoglobulin with the TSA system. DAPI was used for visualization of cell nuclei. Double-positive cells for both αSMA and collagen type 1 were counted as SPC and quantified per 200-fold magnification high power field (HPF). Isotype-stained sections served as controls.

To verify that spleen mononuclear cell giving rise to SPC in culture were indeed bone marrow derived cells and not spleen stroma, we performed a control experiment in which we transplanted bone marrow from GFP-mice to lethally irradiated animals. After peripheral blood chimerism was confirmed, two of four animals received STZ to induce diabetes. Similar to our main study protocol, after 8 weeks diabetes, spleen mononuclear cells were isolated, analyzed for GFP-expression on flow cytometry, and placed in SPC-culture. After 7 days in culture, SPC were detached using trypsin/EDTA and analyzed for GFP-expression on flow cytometry.

### MAC-3 staining

To identify monocyte/macrophages, sections from GFP-chimeric animals and paraformaldehyde-fixated SPC on cover slips were stained with rat-anti-mouse MAC-3 antibody (BD Pharmingen, San Diego, USA), which was visualized using a TRITC-labeled goat-anti-rat secondary antibody (Serotec, Hilversum, the Netherlands).

### Quantitative real-time PCR for phenotypical conformation of αSMA, collagen-I and calponin expression and quantification of TGF-β and BMP6 mRNA expression

Expression of αSMA, collagen-I and also calponin was verified using real-time PCR. For this, total RNA was extracted from SPC with RNeasy columns (Qiagen, Venlo, The Netherlands) according to the manufacturer's instructions. One microgram of total RNA was reverse transcribed to cDNA using oligo-dT, random hexamers, and Superscript reverse transcriptase (Invitrogen, Carlsbad, CA, USA). Taqman quantitative real-time PCR reactions were performed in duplicate on an ABI Prism 7700 Sequence Detection System using pre-designed primer sets for of αSMA, collagen-I, calponin, TGF-β1, BMP-6, and housekeeping gene β-actin (Taqman assays-on-demand, Applied Biosystems, Nieuwerkerk a/d IJssel, The Netherlands). mRNA expression was quantified using the comparative Ct method using β-actin as the reference gene. β-actin was chosen as reference gene as it has been shown to be a stable housekeeping gene in diabetic conditions in vitro and in vivo [11].

### Statistical analysis

All data are presented as mean ± SEM. The Mann-Whitney test was used to compare means between groups. A value of p < 0.05 was considered statistically significant.

## Results

### Course of diabetes

Glucose levels exceeded twice the upper limit of normal within 2 days after STZ injection. Blood glucose levels in diabetic mice remained at least above twice the upper limit of normal during the course of the experiment (average 24.5 ± 1.6 mmol/l). Blood glucose levels in control mice ranged from 6.5 to 9.5 mmol/l (average 8.5 ± 0.5 mmol/l; p < 0.001 vs diabetic mice). At termination after a total of 8 weeks hyperglycemia, HbA1c levels were 8.3 ± 0.4% in STZ diabetic mice compared to 4.9 ± 0.5% in control mice (p < 0.0001). After injection with streptozotocin, diabetic mice initially lost some weight, but this stabilized after insulin pellet placement. During the further study protocol body weights remained fairly constant in diabetic mice (25.2 ± 0.7 vs. 23.7 ± 1.3, p = ns), while there was normal weight gain in control mice (24.9 ± 0.7 vs. 35.1 ± 1.0, p < 0.0001).

### Intimal hyperplasia is exaggerated after cuff-induced vascular injury in diabetic mice

**Figure 1:**
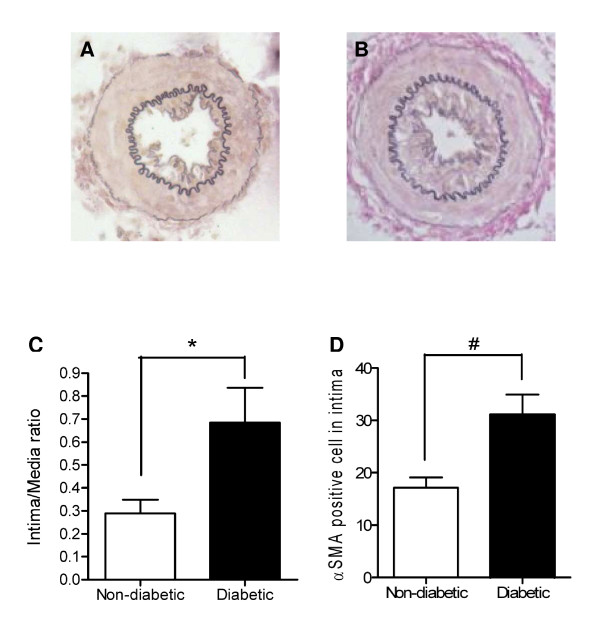
**Intima formation in cuffed femoral artery segments**. Representative pictures of Elastin Von Gieson stained cuffed femoral artery sections from non-diabetic (A) and diabetic animals (B). 21 Days after vascular injury intima/media ratio is higher in diabetic animals (C) and intimal lesions of diabetic animals contain more aSMA positive cells as compared to control animals (D). *p < 0.05, # p < 0.005

We evaluated if the STZ-induced diabetic mice in our study displayed an exaggerated neointima formation as we expected. Indeed, twenty-one days after vascular injury the intima-media ratio was higher in diabetic animals than in controls (0.68 ± 0.15 versus 0.29 ± 0.06; p < 0.05, Figure 1), corresponding with an increased number of intimal αSMA-positive cells (31 ± 4 vs 17 ± 2 cells per cross-section; p < 0.005. Figure 1). No intima hyperplasia was observed in non-cuffed control arterial segments of both control and diabetic animals.

### Bone marrow derived cells home to sites of neointima formation

**Figure 2:**
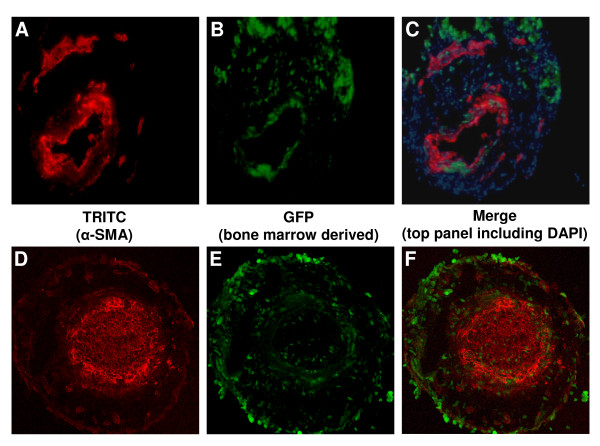
**GFP-positive bone marrow derived cells incorporate into the vessel wall at sites of neointimal formation**. Sections from cuffed femoral artery segments from GFP-chimeric animals show incorporation of bone marrow derived cells. TRITC-labeled α-SMA-positive and bone marrow derived GFP-positive cells are shown in separate fluorescence channels (A/D and B/E respectively) and in overlay images (C/F, including DAPI for C). Panels A/B/C show representative pictures using a regular fluorescence microscope, which was used to quantify GFP-positive cell incorporation (G). Substantial numbers of GFP-positive cells incorporated into the cuffed femoral artery segment, particularly in the adventitia (G). Panels D/E/F show a striking example of a section of a cuffed arterial segment that is completely occluded by neointima, which has been visualized using a confocal fluorescence microscope. A detail of the merged picture F is shown in H, illustrating that most of the bone marrow derived GFP-positive cells are located in the adventitia, although various GFP-positive cells can be found dispersed throughout the neointima.

**Figure 3:**
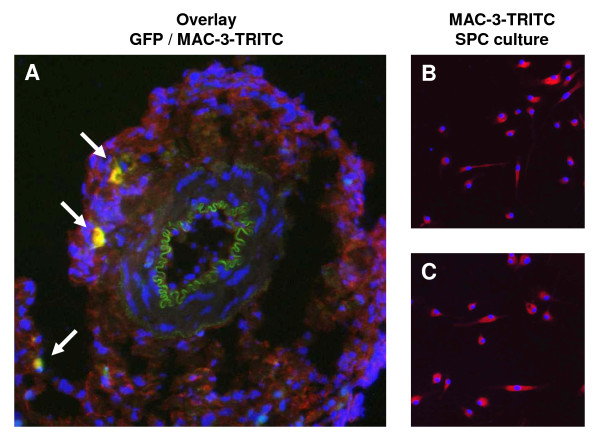
**MAC-3 expression by adventitial bone marrow derived cells and SPC in culture**. Adventitial GFP-positive bone marrow derived cells were frequently positive for macrophage marker MAC-3 (white arrows; A). SPC from both control (B) and diabetic (C) mice were uniformly MAC-3 positive.

In cuffed arterial segments of GFP-chimeric animals, GFP-positive bone marrow derived cells were observed in the neointima (4.4 ± 3.3 cells per section) and particularly in the adventitia (43.6 ± 9.3 cells per section). These cells were in majority α-SMA negative (Figure 2), but frequently positive for macrophage marker MAC-3 (Figure 3A). In non-cuffed control arterial segments bone marrow derived cells were observed only sporadically in the adventitia.

### SPC differentiation is accelerated under diabetic conditions

**Figure 4:**
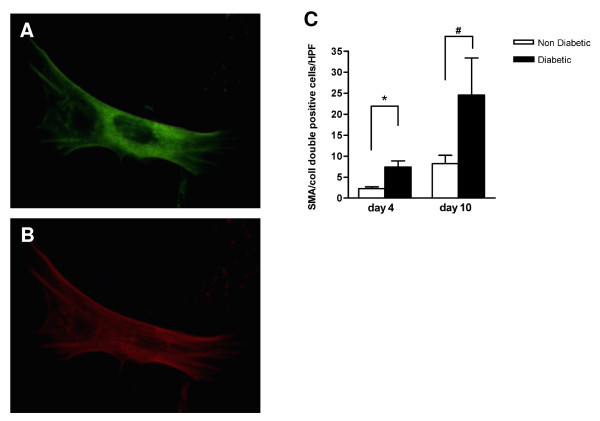
**SPC characterization and quantification**. Cultured mouse SPC expressed collagen type I (detected by FITC-labelled antibody; A) and α-smooth muscle actin (detected by TRITC-labeled antibody; B) demonstrated by immunofluorescent staining. The number of SPC obtained after 4 and 10 days culture (C) is higher in diabetic animals (black bars) than in non-diabetic animals (white bars). * p < 0.01, # p < 0.05

Under SPC culture conditions, a proportion of spleen mononuclear cells became adherent to the culture dish, adopted an elongated morphology and started expressing α-SMA and collagen type 1 (Figure 4AB). After 4 days of culture, 20 ± 6% of adherent cells from control animals were double-positive for both α-SMA and collagen type 1 and were thus identifiable as SPC, while more than 50 ± 4% of adherent cells had differentiated into SPC in cultures from diabetic animals (p < 0.001, Figure 4C). After 10 days of culture, nearly 100% of adherent cells was positive for SPC markers in cultures from both diabetic and control mice. Real-time PCR confirmed the expression of αSMA, collagen-I and also calponin. SPC from both control and diabetic mice were negative for endothelial marker CD31, but uniformly positive for macrophage marker MAC-3 (Figure 3BC).

### SPC number is increased in diabetic mice

The absolute number of SPC cultured from diabetic animals after 4 days was higher than from controls (7.5 ± 1.4 vs 2.3 ± 0.4 per HPF; p < 0.01, Figure 4C). After 10 days, total cell number in both groups had increased further than the original number of adherent cells on day 4, indicating that SPC proliferate in culture. The total number of SPC in cultures from diabetic mice remained substantially higher compared to cultures from controls (24.6 ± 8.8 vs 8.3 ± 2.0 per HPF; p < 0.05, Figure 4C)

### SPC cultured from spleen mononuclear cells are bone marrow derived

A control experiment in GFP bone marrow transplanted mice showed that indeed the vast majority of mononuclear cells isolated from the spleen are bone marrow derived (range: 91-94% GFP-positive, n = 4). Also after culturing the spleen mononuclear cells to SPC, most proved bone marrow derived in both control and diabetic animals (range 79-91%), indicating that contaminating spleen stroma is at most a minor factor in SPC cultures from spleen.

### mRNA expression of BMP6 is decreased and of TGF-β increased in diabetic SPC

**Figure 5:**
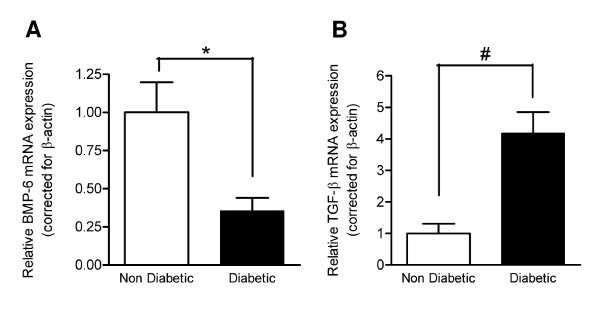
**BMP-6 and TGF-β mRNA expression in SPC**. Quantitative PCR showed decreased BMP-6 (A) and increased TGF-β (B) mRNA expression in SPC from diabetic animals compared to controls. mRNA levels are expressed relative to the mean expression in the non-diabetic control group in SPC cultured for 10 days. * p < 0.05, # p < 0.001

Quantitative RT-PCR of SPC cultured for 10 days showed a 2.7-fold down regulation of BMP-6 in SPC derived from diabetic mice in comparison with control SPC (p < 0.05, Figure 5A). TGF-β expression in diabetic SPC was 4.2-fold up regulated compared to control SPC (p < 0.001, Figure 5B).

## Discussion

In the present study, we show increased frequency of SPC in peripheral blood cultures from diabetic mice, associated with an increased expression of TGF-β and decreased expression of BMP-6 in the diabetic SPC. This modulation of TGF-β/BMP-6 expression may underlie enhanced SPC differentiation and expansion from diabetic mononuclear cells *ex vivo *and may be of consequence for expanding intimal lesions to which SPC home. In GFP-chimeric animals we show that substantial numbers of bone marrow derived cells incorporate into sites of neointimal formation, particularly the adventia. Mouse SPC expressed MAC-3, which would be consistent with a monocytic origin. Bone-marrow derived cells in the vessel wall of GFP-chimeric animals in part expressed MAC-3, but we rarely observed α-SMA expression in GFP-positive cells.

We observed increased neointima formation in STZ-induced diabetic mice, while intimal hyperplasia was not increased in STZ-induced diabetic rats in two earlier studies[12,13]. This may be related to differences in the animal model of arterial injury as we used a non-constrictive perivascular cuff, where the other two studies used balloon denudation of the carotid artery and placement of an aortic bare metal stent[12,13]. Perivascular inflammation is pivotal in the non-restrictive cuff model[14]. We chose the non-restrictive cuff model for its good reproducibility[15] and because it results in modest intimal hyperplasia and incorporation of bone marrow derived cells under control conditions[3], while other models induce such strong intimal hyperplasia in controls that aggravating influences may be obscured.

Using bone marrow chimeric animals, we show that part of the neointimal cells are of bone marrow origin, consistent with recent reports by others[3]. It was shown that smooth muscle cells of bone marrow origin also contribute to transplant arteriosclerosis and hyperlipidemia-induced atherosclerosis[16-19]. Histological studies of autopsy material of patients after bone marrow transplantation confirmed that smooth muscle cells in human vascular lesions are in part bone marrow derived[20]. The origin of SPC, their regulation, and how SPC respond to pathophysiological stimuli associated with the development of CVD has not been fully elucidated. One study found SPC to be mainly derived from CD34+ hematopoietic stem cells, but others found circulating endoglin(CD105)+ CD14+ monocytes as a main source of SPC[8]. Interestingly, circulating levels of these CD105+CD14+ cells were increased in a population of mostly non-diabetic patients with manifest CVD compared to controls, suggesting that SPC levels may be increased in a pro-atherosclerotic milieu[8]. We have previously reported increased SPC differentiation in Type I diabetic patients, in which we cannot exclude an influence of a presence of subclinical atherosclerotic disease[9]. Our current observation of enhanced SPC levels in mice with inducible diabetes provides strong additional evidence for the involvement of diabetes in enhancement of SPC differentiation. The uniform expression of macrophage marker MAC-3 by SPC supports that these cells are of monocytic origin. Although we found MAC-3 positive bone-marrow derived cells in the vessel wall of cuffed arterial segments as a component of the perivascular inflammatory infiltrate, we rarely observed α-SMA-expression in incorporated bone marrow derived cells. Therefore, adventitial MAC-3 positive cells do not necessarily undergo mesenchymal differentiation, even in a pro-fibrotic environment. This observation is in line with a previous study, in which bone marrow derived adventitial MAC-3-positive cells did not show clear α-SMA-expression in intimal hyperplasia in a cuff model[21]. Interestingly, MAC-3 positive cells were shown to express histidine decarboxylase (HDC) and intimal hyperplasia was attenuated in HDC knockout animals, suggesting a relevant pathophysiological role mediated by histamine[21]. Importantly, plasma histamine levels and HDS activity in various tissues are increased in diabetes[22].

BMP-6 may act as both a stimulator and suppressor of epidermal proliferation[23]. Over-expressing BMP-6 delays scar formation in wound healing[24], but stimulates fibrous encapsulating of BMP-6 over-expressing tumours[25]. TGF-β enhances the outgrowth of SPC-like collagen-secreting pericytes from mononuclear cell cultures[26]. Under these circumstances, BMP-6 may act as an inhibitor of TGF-β and BMP-6 was shown to attenuate TGF-β-induced upregulation of a variety of SMC differentiation markers, including smooth muscle actin, in mature smooth muscle cells[27]. Diabetes is associated with higher TGF-β levels in the circulation[28,29]. Therefore, we assessed the expression TGF-β and BMP-6 in SPC from diabetic animals. We observed increased TGF-β and decreased BMP-6 expression in diabetic SPC, which could explain the accelerated maturation and increased frequency of diabetic SPC in culture. Furthermore, since circulating SPC home to sites of injury and constitute a significant proportion of neointimal cells[3,18] and in our observations also a major source of adventitial cells, enhanced TGF-β and reduced BMP-6 production may exert paracrine effects on resident smooth muscle cells. Overexpression of TGF-β induces formation of a cellular and matrix-rich intima even in uninjured arteries[30], while inhibition of TGF-β signalling attenuates intimal hyperplasia and remodeling after vascular injury[31,32]. We indeed observed aggravated intima-formation in the diabetic mice in our study. As we observed low numbers of bone marrow derived cells incorporating into the neointima and only rarely α-SMA expression in these cells, we speculate that the paracrine factors from the abundant adventitial bone marrow derived cells are the predominant mode of influence.

Interestingly, in both our current animal study and the previously reported study in human patients with type I diabetes, augmented SPC differentiation and sustainably increased SPC proliferation was observed ex vivo under normoglycemic culture conditions. This indicates that the effects of the hyperglycemic state in vivo are to some extent 'imprinted' upon the circulating progenitor cell population. Further research is required which mechanisms are involved in this process.

In this study we used spleen mononuclear cells because of the low numbers of circulating cell numbers in mice. This is different from our previous human study in which SPC were isolated from peripheral blood[9]. Spleen mononuclear cells are a reservoir for circulating mononuclear cells and permit higher cell numbers to be obtained from individual animals. Using GFP-bone marrow transplanted animals, we confirmed that cultured splenic SPC are indeed from hematological lineage.

Our study has several limitations. We used an inducible model of diabetes with STZ, which allows for the use of excellent controls, but could be confounded by non-diabetic toxic effects. However, as STZ has a short half-life of 1 hour after intraperitoneal injection in mice and our assessment of the developing neointima and cell cultures took place 8 weeks after STZ injection, direct toxic effects of STZ on cultured cells or the developing neointima are highly unlikely and we are confident that the effects are mediated by the hyperglycaemic state of the animals. Identification of SPC required *in vitro *culture for several days as a-SMA and/or collagen-I expression could not be detected by immunocytochemistry on freshly isolated cells. Therefore, we cannot determine if the numeric differences observed by us in SPC cultures of diabetic animals after 4 days was the result of an increased level of circulating SPC or if it is attributable to enhanced proliferation or survival *in vitro*. Also, we were unable to reliably quantify TGF-β and BMP-6 protein levels in the cultured SPC and thus did not exclude if posttranscriptional modulation may have attenuated the effects of the presence of diabetes on TGF-β and BMP-6 gene expression. In vivo, definite confirmation of modulation of TGF-β/BMP-6 expression in the neointima itself could not be confirmed by us and remains to some extent speculative. Other studies do confirm increased TGF-β in arteries of diabetic animals in vivo[33-36].

The present data are consistent with our previous observations that SPC levels are increased in Type I diabetic patients and lack BMP-6[9]. Increased SPC numbers have also been observed in (non-diabetic) patients with manifest coronary artery disease[8]; however, whether SPC from these patients have altered TGF-β/BMP-6 expression has not been studied.

## Conclusions

The current study shows that in a diabetic mouse model, SPC levels are increased and SPC TGF-β/BMP-6 expression is modulated. Altered TGF-β/BMP-6 expression is known to regulate smooth muscle cell differentiation and may facilitate SPC differentiation. This may contribute to exaggerated intimal hyperplasia in diabetes as bone marrow derived cells home to sites of neointima formation. Further studies are required to evaluate if inhibition of SPC differentiation, e.g. by modulating the TGF-β/BMP-6 expression, may reduce intimal hyperplasia in diabetes.

## Competing interests

The authors declare that they have no competing interests.

## Authors' contributions

PEW and MCV conceived the study. PEW and CTJV performed animal experiments, histology, cell culture, molecular analysis, data preparation, statistical analysis and drafted the manuscript. KO performed animal experiments and histology. TQN contributed to molecular analysis and advised on cell culture. DPVK, RG and MJG advised on study setup, data interpretation and manuscript preparation. MCV participated in the design of the study, interpretation and analysis of data, and helped draft and revise the manuscript. All authors read and approved the final manuscript.
